# The Impact of Upstream Sub-Basins’ Water Use on Middle Stream and Downstream Sub-Basins’ Water Security at Country-Basin Unit Spatial Scale and Monthly Temporal Resolution

**DOI:** 10.3390/ijerph16030450

**Published:** 2019-02-03

**Authors:** Dagmawi Mulugeta Degefu, Zaiyi Liao, Weijun He, Liang Yuan, Min An, Zhaofang Zhang, Wu Xia

**Affiliations:** 1 College of Economics and Management, China Three Gorges University, Yichang 443002, China; dagmawi.degefu@ryerson.ca (D.M.D.); weijunhe@ctgu.edu.cn (W.H.); anmin@hhu.edu.cn (M.A.); 2 Faculty of Engineering and Architectural Science, Ryerson University, Toronto, ON M5B 2K3, Canada; zliao@ryerson.ca; 3 School of business, Hohai University, Nanjing 210098, China; zackzhang@hhu.edu.cn; 4 School of Law and Public Administration, China Three Gorges University, Yichang 443002, China; ctguwuxia@163.com

**Keywords:** Transboundary river basin, Water stress, River discharge, Water footprint, Water dispute

## Abstract

Water, in most of the transboundary river basins, is a bone of contention among their riparian states. Taking this into account, this article assessed the monthly impact of upstream water withdrawal on the water security of middle stream and downstream sub-basins at a country-basin mesh spatial resolution. Roughly 2.18 billion people in 442 sub-basin areas experience water stress intensification by less than 1% throughout the year. In addition, 2.12 billion people in 336 sub-basin areas experience water stress level change, from no water stress to one of the water stress categories, for at least one month as the result of upstream withdrawal. Even though there is a clear upstream impact in many of the basins, water disputes with severe social, economic, political, and environmental consequences are nonexistent. This might be an indication that grave water disputes are the result of complex socio-economic and political interactions, not merely because of water deficits due to upstream water withdrawal. Therefore, understanding this relationship is crucial in identifying inflection points for water conflicts within transboundary river basins.

## 1. Introduction

Water is one of the most important natural resources, and is crucial for maintaining socioeconomic and environmental systems [1,2]. Hence, ensuring water security by maintaining the quality and quantity of water required for human consumption and environmental integrity is very critical. Most of these water resources stretch across a large part of the earth’s surface, creating socioeconomic and environmental inter-dependencies among their upstream, middle stream, and downstream sub-basins. Therefore, the quantity of available water within the middle stream and downstream sub-basins depends on water withdrawal and climatic patterns in relevant upstream sub-basins. Furthermore, the quality of the rivers’ water capital is another important element affecting water security which varies through time and space.

Transboundary basins are examples of the freshwater resources with a huge global significance [3,4,5,6,7]. In transboundary basins, upstream, middle stream, and downstream sub-basins at a country-basin mesh spatial resolution may be under water management regimes that are dissimilar. Water use and natural runoff varies through time and space [8,9]. As a result, the water inter-dependencies among the upstream, middle stream, and downstream sub-basins in these rivers vary through time and space, as well.

There is a current concern that climate change and increasing water footprints are escalating the competition for water in border-crossing basins [5]. Socioeconomic activities and environmental ecosystems could be disturbed under such circumstances, which could lead to water conflicts. Water conflicts, associated with internationally shared water bodies, are generally related to the allocation and use of water resources [6,7,10]. Water quality issues are yet to be attended to in these basins. Even though it is not within the scope of this article, the authors recognize that water quality is one of determining factors of water security. In most of the transboundary river basins, water allocation disputes and negotiations are based on the notions of absolute territorial sovereignty and absolute territorial integrity [11]. The former is promoted by upstream sub-basins, while the latter is the main negotiation strategy for downstream sub-basins. Middle stream sub-basins use both principles as a basis for their water claims. The Nile river basin is a typical transboundary river basin, where the riparian countries claim the river’s water capital based on these principles. These two principles are polarizing strategies for claiming water within border-crossing basins [12]. 

Understanding water availability and footprints throughout time and space is very crucial in designing sustainable water allocation and use policies [8,9]. Water management researchers have become infatuated with understanding water availability and footprints on various spatial and temporal scales [3,4,5,9,13,14,15] These studies varied in the way they quantified water availability, water footprint, and environmental water requirements [9]. In these studies, water scarcity is usually quantified as the ratio of water demand to available water (demand-driven water scarcity), or as per-capita water availability (population-driven water scarcity).

Considering their socio-economic environmental importance, and the fact that their water capital is key in the contention among their sovereign riparian states, studies have been carried out to quantify water availability and footprints exclusively within transboundary basins. Munia et al. [5,16], Degefu et al. [9], and Wada and Heinrich [17] are among the most recent studies done on a global scale. These studies computed surface water runoff and water use on a spatial mesh formed by overlapping national borders on basin boundaries. This is a more practical spatial scale for measuring water stress [9]. The study by Wada and Heinrich [16] was solely focused on transboundary aquifers, while Degefu et al. [9] focused their study on potential water demand rather than actual water withdrawal, which is independent of the available water. In addition, their study failed to show the impact of upstream water use on the middle stream and downstream sub-basin water security. Studies by Munia et al. [5,16] quantified the biennial impact of upstream water use on the availability of water in the middle stream and downstream country-basin units of transboundary river basins on a global scale. Munia et al. [5] found that, as a result of water withdrawing socioeconomic activities in upstream sub-basins, water stress level increased in 30–65 sub-basins of border crossing basins, impacting 0.29–1.13 billion people annually. In their continued work, Munia et al. [16] categorized country-basin units of transboundary river basins into different kinds of upstream water dependency at an annual temporal resolution. However, socioeconomic activities and climatic patterns are characterized by seasonal variations. As a result, the impact of upstream water use on the water security of middle stream and downstream sub-basins within transboundary river basins was also seasonal [18,19,20]. Therefore, even though the studies by Munia et al. [5,16] were done at country-basin spatial resolution, the annual time scale was not fine enough to take these variations into account. As a result, annual studies might underestimate or overestimate the impact of upstream sub-basin water use on middle stream and downstream sub-basin water security. 

To address this research gap, in this article the monthly impact of upstream water withdrawal on middle and downstream sub-basin water security is computed at a country-basin spatial resolution. The difference between water stress level, calculated using the principles of absolute territorial integrity and absolute territorial sovereignty, portrays the impact of the upstream water use on the middle stream and downstream sub-basin water scarcity intensity.

## 2. Materials and Methods 

Water use data was obtained from Wada et al. [21]. These values were determined by dynamically integrating water availability and demand models to simulate the actual water withdrawal [21]. The water withdrawal data was taken and aggregated for the year 2010, instead of the average for a period of years, in order to ensure that the rise in water demand through the years is not hidden, which might be if the data average is used.

**Figure 1:**
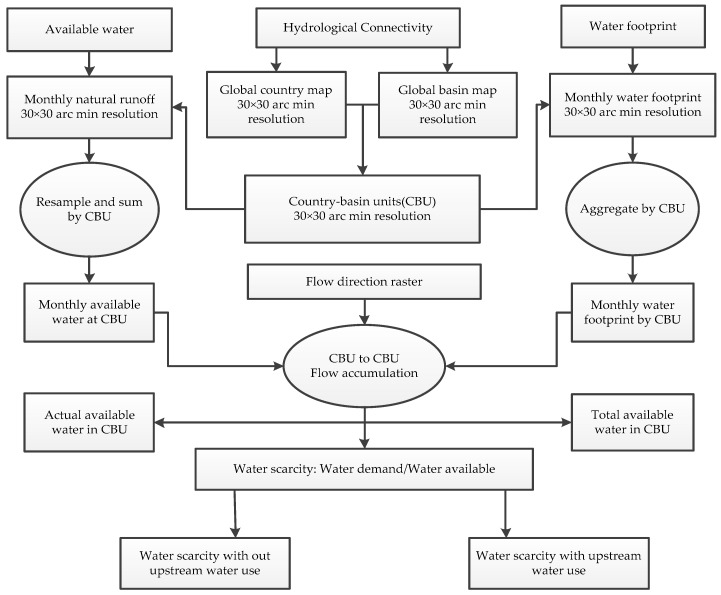
A methodological framework for determining the impact of upstream sub-basin water footprint on downstream sub-basin water security.

Water availability was calculated by averaging the monthly river discharge for the period from 1958–2010 and aggregating the data obtained a country-basin spatial resolution. To compute upstream water withdrawal, the water use data was routed from upstream to downstream. Flow direction data from Fekete et al. [22] was used to route the water footprint from upstream to downstream at a spatial resolution of 30 arc minutes (see Figure 1). 

Only one-fifth of the available water was quantified based on the principle of absolute territorial integrity, and absolute territorial sovereignty was assumed for the water accessible for withdrawal in each sub-basin. The rest was presumed to be allocated as a cautionary amount for maintaining the integrity of the river aquatic ecosystem. This assumption is based on the study by Richter et al. [23]. The population count for the study year was obtained from the Center for International Earth Science Information Network (CIESIN) [24].

Water scarcity was classified according to Munia et al. [5]. Country-basin units with water stress values of less than 0.2% are categorized as under no water stress. Sub-basins with water stress values between 0.2–0.4% are classified as to be under moderate water stress. Those sub-basins with water stress values between 0.4–0.7% are grouped as under high stress. Country-basin units with water stress values above 0.7% are clustered to be under extreme water stress. River sub-basins with negative water stress values only have the local natural runoff (minus the amount of water allotted for ecosystem maintenance) as water available for withdrawal. The water scarcity intensification in the middle stream and downstream river sub-basins as the result of the upstream water footprint are categorized into the following groups: (1) 0%–1%, (2) 1%–5%, (3) 5%–10%, (4) 10%–20%, and (5) >20%.

## 3. Results and Discussion

### 3.1. Water Stress under Absolute Territorial Integrity

The results obtained in this study showed that the numbers of people experiencing moderate, high, and extreme water stress at least for a month are 1.23, 1.67, and 1.76 billion respectively (see Table S1 and Table S2). The numbers of people living under similar water stress levels for three to six months within a year are 722, 482.54, and 295.24 million. These people dwell within 239,196 and 206 country-basin units, respectively (see Figure S1). Only 35 sub-basins containing 311.33 million people face extreme water stress throughout the year. The results obtained in this study differ slightly from those done in recent years at the same spatial resolution. Considering the disparities among the studies, in terms of water availability metric, water footprint metric, and temporal resolution, these differences are reasonable.

**Figure 2:**
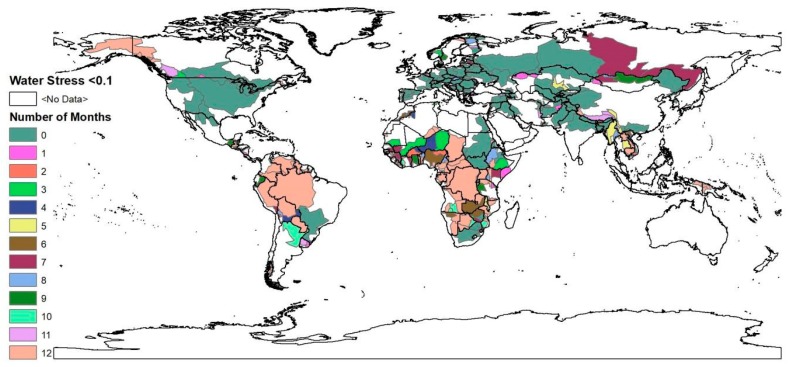

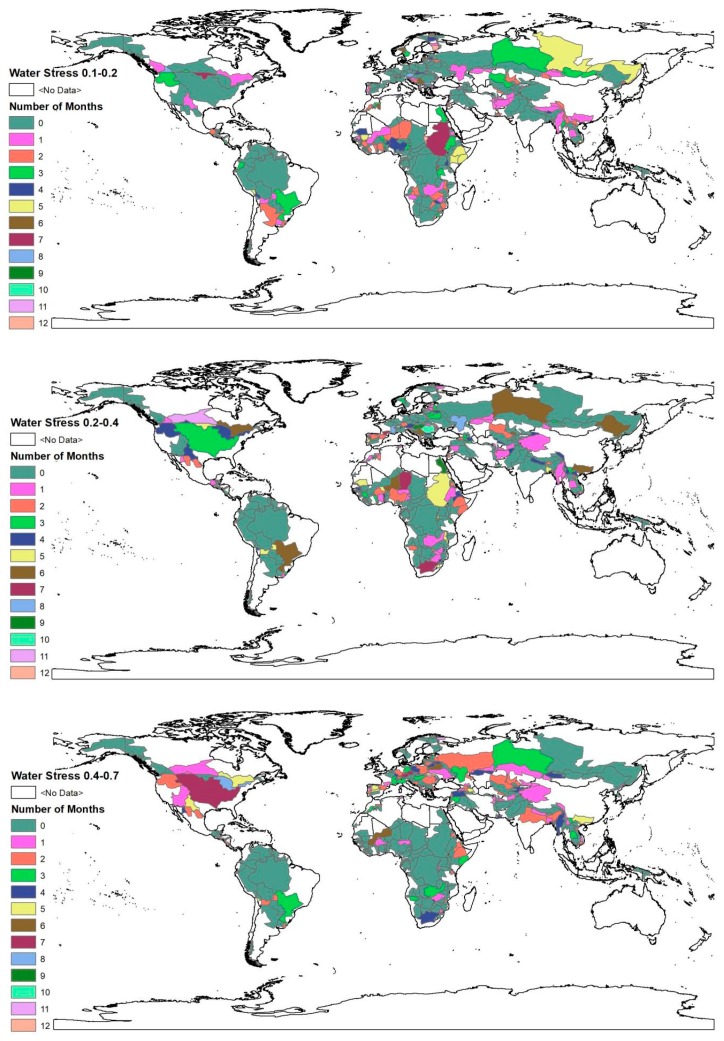

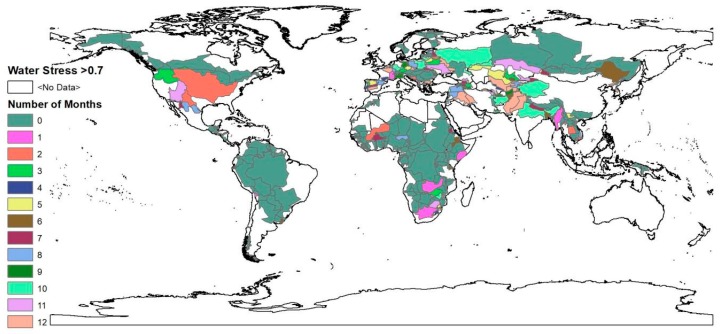
The number of months each sub-basin spends under different levels of water stress. This map was generated with ArcGIS 10.2 for desktop from Environmental Systems Research Institute (ESRI) [25].

Most of the sub-basins identified as being under water stress by Degefu et al. [9] match those indicated in this article (see Figure 2). These river basins are in North & West Africa, the Middle East, Europe, the Middle of the United States, and South-east Asia. Among the sub-basins in these regions the main ones are the Colorado & Rio Grande river sub-basins in the United States & Mexico, the Nile in Egypt, the Limpopo river’s country-basin units, Lake Chad & the Niger rivers’ sub-basins in Algeria, the Ganges-Brahmaputra-Meghna sub-basins in India & Bangladesh, country-basin units of the Aral Sea, the Tigris-Euphrates/Shatt al Arab river country-basin units, the Tarim river’s sub-basin in China, and the Tagus/Tejo & Guadiana country-basin units in Spain. These basins are characterized either by low annual river discharge, high population count, or large-scale water intensive socioeconomic activities.

The above results concur with the fact that water scarcity is mainly due to temporal and spatial mismatch of available water & water footprint. Hence, quantifying water scarcity at a seasonal temporal scale provides a better picture of the problem. The number of people experiencing different levels of water scarcity is not highly sensitive to the available water and water footprint metrics.

Even though the number of people living under water stress is significant, the majority of these people dwell within 48 country basin units. Each of these sub-basins contains from 10 million up to half a billion people, and accounts around 71% of the total population within the transboundary river basins. The Ganges-Brahmaputra-Meghna sub-basins in India & Bangladesh are typical examples of highly populous country-basin units. If water scarcity is computed as population-driven water scarcity, the number of people experiencing different levels of water scarcity is expected to be highly sensitive to the water availability and footprint metrics.

### 3.2. Water Stress Intensification as the Result of Absolute Territorial Sovereignty

**Figure 3:**
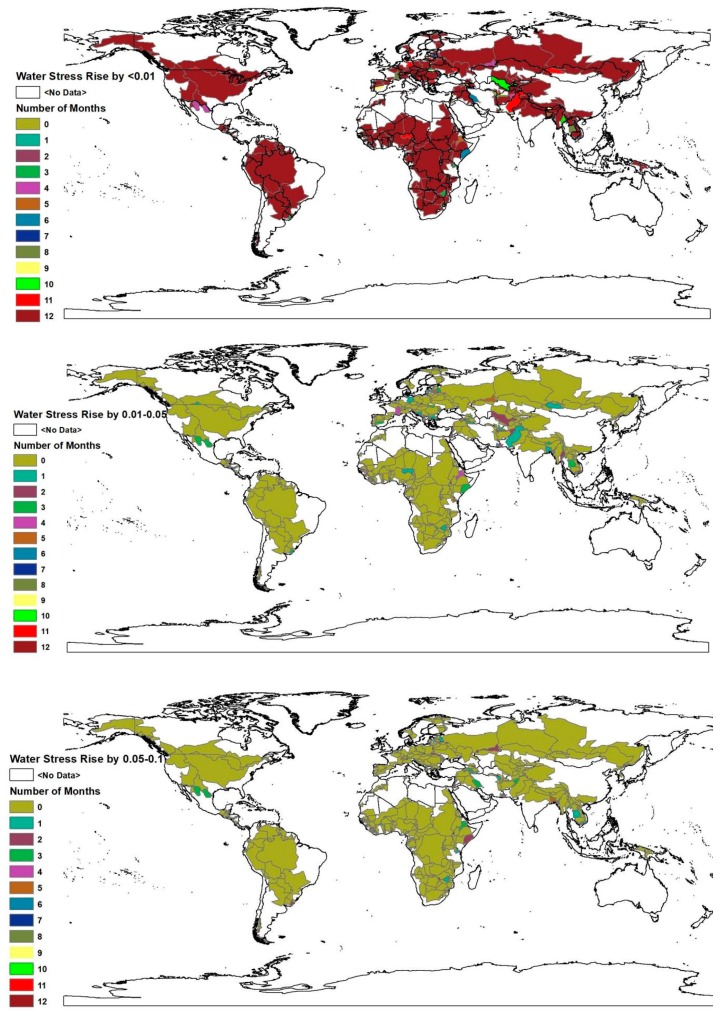

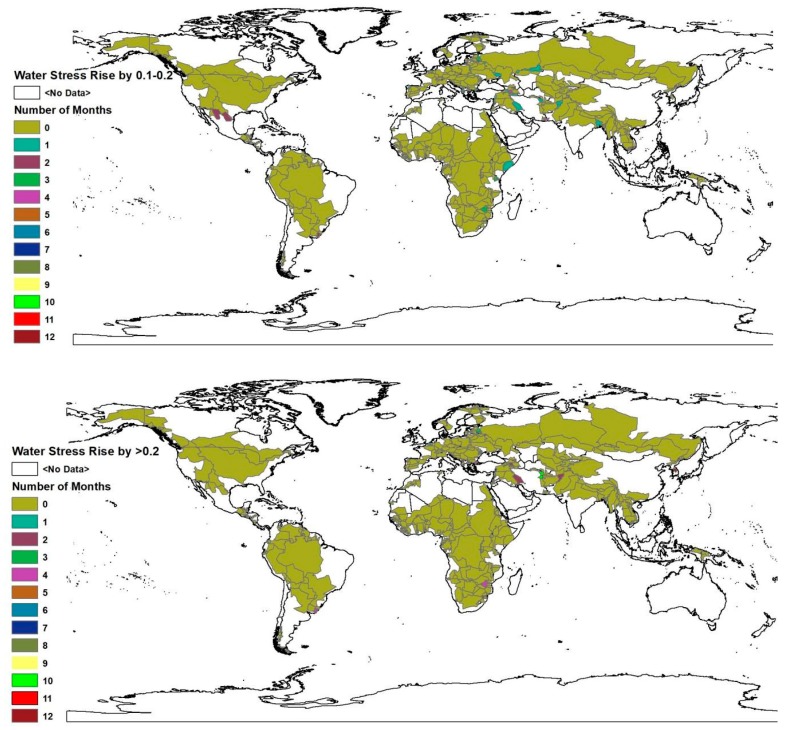
The number of months in which each sub-basin experiences different levels of water stress intensification. This map was generated with ArcGIS 10.2 for desktop from Environmental Systems Research Institute (ESRI) [25].

This article acknowledges the fact that the impact of upstream sub-basin water footprint on the water security of middle stream and downstream sub-basins varies intra-annually. Hence, it is identified in the sub-basins and the number of people whose water stress level intensifies, as the result of upstream water withdrawal, is estimated at a monthly time step (see Figure 3). The number of country-basin units and people which experience different levels of water stress intensification are shown in Table 1, below.

Approximately 80.4% of the total population within transboundary river basins experience water stress intensification by less than one percent throughout the year (see Figure S2 and Table S3). The number of people facing water stress intensification, at least for a month, by 10%–20%, due to upstream water withdrawal, is 207.43 million. Those sub-basins that experience water stress level escalation by more than 20%, due to upstream water use, at least for one month are 55. These sub-basins have 77.87 million people. Only 13.9 million people within three sub-basins experience a rise in water stress level by more than 20%, as the result of the upstream water footprint throughout the year. These sub-basins are the Hanx in South Korea, the Cullen in Chile, and the Daugava in Latvia. No change in water stress level is observed for the upstream country-basin units. In addition, the number of country-basin units whose water stress category changes from no water stress to water stress, as the result of upstream water use, for at least a month, is 336. These basins contain 2.11 billion people. Around one billion, 187 million people within 237 and 61 country-basin units experience the same shift, due to upstream water withdrawal, for at least three and six months, respectively.

The results obtained in this study showed that there was an obvious impact on the middle stream and downstream country-basin units’ water security, as a result of upstream water withdrawal. If so, were there serious water disputes with huge socio-economic and environmental consequences in the past? Or, do such water conflicts exist now? There have been reported incidences of water disputes within transboundary river basins [26,27]. Furthermore, some river basins are identified as being at greater risk of experiencing water conflict [28]. However, serious water conflicts with severe social, economic, political, and environmental consequences are yet to occur in most of the transboundary river basins. This might be because of the following main reasons. First, in most of the transboundary basins, each riparian state’s right to have access to a portion of the transboundary water body’s water is acknowledged by the upstream, middle stream, and downstream riparian states. Existing international water management conventions also recognize this right [29]. This decreases the sensitivity of most of the middle and downstream sub-basins to upstream water use, to a certain extent. This is true, particularly in border-crossing basins with minimal upstream water withdrawals, water-sharing agreements, or ongoing negotiations to link issues or to allocate the water capital. The Nile river basin is a typical example. Next, in most of the river basins, monthly water stress intensification as the result of the upstream water footprint is less than one percent for most of the year. This might not be difficult to adjust through demand management techniques, or by alternative water resources (groundwater or water transfer projects). Additionally, for some sub-basins, even though they are in the middle and downstream sections of the river basins, their water stress levels are barely intensified. This could be either because upstream water use hardly exists, or a significant portion of the river discharge originates within them. For instance, in the Nile river basin, there are two main tributaries (The Blue and White Nile) with very distinct features. These features explain different upstream-downstream relationships. The Blue Nile is the source of more than 80% of the basin’s water. The upstream consumption in this tributary is minimal, hence it is yet to affect the water security of the sub-basins in the downstream countries. On the other hand, the White Nile is not the major water contributor to the basin total discharge; as a result, the upstream agricultural consumption is not significant enough to affect the availability of water to the middle stream and downstream sub-basins. Finally, in some sub-basins, even though the water stress increases significantly as the result of the upstream water footprint, water disputes were not reported. This could be the case either because the rivers’ socioeconomic significance was minimal, or alternative water resources were available within the sub-basins. These might be the reasons why, even though many of the sub-basins at a country-basin spatial resolution experience water stress increase as the result of upstream water withdrawal, serious water disputes with significant socioeconomic and political consequences barely exist. Even in some of the river basins, water stress seems to be leading riparian countries into cooperation rather than conflict [28].

This work has the following drawbacks, which need to be considered when interpreting the results. First, a general assumption was made when quantifying environmental water requirements. Environmental requirements needed to be individually quantified for each sub-basin, to ensure environmental water needs within each sub-basin is balanced with the water footprint. Secondly, the water stress calculated in this study was demand driven. Hence, care should be taken when interpreting the population numbers living under water stress. Thirdly, the role which water reservoirs play, in terms of regulating the availability of water, may be incorporated into future water stress studies. Finally, seasonal water stress needs to be simulated in these sub-basins, to forecast future socioeconomic and climatic changes. 

## 4. Conclusions

At present, increasing water demand and climate change are intensifying the rivalry over water within transboundary river basins. Considering the global significance of such basins, this article provided a glimpse into the monthly impact of upstream water withdrawal on the availability of water within the middle stream and downstream sub-basins, at a country-basin mesh spatial resolution. 

The results showed that the number of people and country-basin units that experience water stress intensification, as a result of upstream water use, was significant. Most of the middle stream and downstream sub-basins experienced monthly water scarcity level increase as a result of upstream water withdrawal. However, this decrease in the available water was minimal. Some sub-basins experienced significant water shortages, as a result of upstream water withdrawal, but the population included in these sub-basins was a small fraction of the total population in transboundary river basins. 

The following three conclusions can be made from the results. (1) The impact of the upstream water footprint on the downstream water security is not that significant in most of the transboundary river basins river basins, at a monthly time step, for the most part of the target year. (2) Those river basins which are significantly affected are impacted for only a short time, which could be mitigated by water retaining infrastructures and water demand management. (3) Most of the severely impacted river basins also have a small population. These conclusions could be reasons why we are yet to witness water conflicts that have severe social, economic, and political consequences in most of the transboundary river basins. This is a major indication that serious water conflicts are the result of complex and dynamic socio-economic and political interactions, not only owing to water deficit due to upstream water use. Therefore, understanding the dynamics of social, economic, and political inter-dependencies is the key to determining water conflict thresholds within transboundary river basins.

## Figures and Tables

**Table 1:** The number of people (in millions) and the number of sub-basins that experience different levels of water stress intensification due to upstream water withdrawal. The UN adjusted population count for the target year obtained from the Center for International Earth Science Information Network (CIESIN) [24] was used.

Number of Months per Year	Number of People (in Millions) Experiencing Different Levels of Water Scarcity Intensification as the Result of Upstream Water Use					Number of Sub-Basins Experiencing Different Levels of Water Scarcity Intensification as the Result of Upstream Water Use				
	0–0.01	0.01–0.05	0.05–0.1	0.1–0.2	>0.2	0–0.01	0.01–0.05	0.05–0.1	0.1–0.2	>0.2
0	28.6	2233.6	2474.92	2509.13	2638.7	16	468	517	521	516
1	9.34	318.9	54.3	168	3.76	5	37	28	29	12
2	3.59	63.77	23.09	28.11	29.56	7	21	14	9	13
3	6.24	59.9	44.32	7.95	0.73	6	21	8	8	4
4	21.77	26.2	0.35	3.37	7.03	9	10	1	4	5
5	137.68	7.47	119.37	0	2.09	7	6	1	0	5
6	21.4	0.3	0.22	0	9.31	6	3	2	0	3
7	18.24	0.73	0	0	0.9	6	2	0	0	3
8	31.42	0.43	0	0	4.89	9	1	0	0	3
9	19.82	5.28	0	0	0	13	1	0	0	0
10	34.6	0.003	0	0	5.7	15	1	0	0	4
11	199.52	0	0	0	0	30	0	0	0	0
12	2184.34	0	0	0	13.9	442	0	0	0	3
Sum	2716.56	2716.56	2716.56	2716.56	2716.56	571	571	571	571	571

## Supplementary Materials

The following are available online at http://www.mdpi.com/1660-4601/16/3/450/s1, Figure S1: Monthly water stress without upstream withdrawal at country-basin unit spatial resolution. This map was generated with ArcGIS 10.2 for desktop from Environmental Systems Research Institute (ESRI) [25]; Figure S2: Monthly water stress intensification due to upstream withdrawal at a country-basin unit spatial resolution. This map was generated with ArcGIS 10.2 for desktop from Environmental Systems Research Institute (ESRI) [25]; Table S1: Number of people (in millions) and a number of country-basin units facing different levels of water stress. UN-adjusted population count for the target year (2010) obtained from the Center for International Earth Science Information Network (CIESIN) [24] was used; Table S2: Water Scarcity Without Upstream Withdrawal; Table S3: Water Scarcity with Upstream Withdrawal Minus Water Scarcity Without Upstream Withdrawal and Number of Months within the Year a Sub-basin Changes Water Stress Category Because of Upstream Water Use.
