# Supplementary material for: The Impact of Upstream Sub-Basins’ Water Use on Middle Stream and Downstream Sub-Basins’ Water Security at Country-Basin Unit Spatial Scale and Monthly Temporal Resolution
Source: Int J Environ Res Public Health. 2019 Feb 3;16(3):450. doi: 10.3390/ijerph16030450 (PMC6388158; doi:10.3390/ijerph16030450)
Supplement: Supplementary file 1 [file ijerph-16-00450-s001.zip › ijerph-430587 - supplementary -proofed/Supplementary Material.docx]

# **Supplementary Material**

**Table S1.** Number of people (in millions) and a number of country-basin units facing different levels of water stress. UN-adjusted population count for the target year (2010) obtained from the Center for International Earth Science Information Network (CIESIN)17 was used.

| **Number of Months**  **Per Year (*n*)** | **Number of People (in Millions) Facing Low, Moderate, Significant and Severe Water Scarcity during *n* Months Per Year** | | | | | **Number of Country-Basin Units Facing Low, Moderate, Significant and Severe Water Scarcity during *n* Months Per Year** | | | | |
| --- | --- | --- | --- | --- | --- | --- | --- | --- | --- | --- |
|  | **0–0.1** | **0.1–0.2** | **0.2–0.4** | **0.4–0.7** | **>0.7** | **0–0.1** | **0.1–0.2** | **0.2–0.4** | **0.4–0.7** | **>0.7** |
| 0 | 1953.41 | 1934.7 | 1484.02 | 1051.17 | 953.45 | 192 | 339 | 335 | 378 | 368 |
| 1 | 27.15 | 249.34 | 245.51 | 323.65 | 77.29 | 22 | 67 | 71 | 57 | 15 |
| 2 | 59.11 | 122.18 | 148.73 | 734.59 | 128.55 | 12 | 61 | 42 | 47 | 17 |
| 3 | 34.76 | 1191.97 | 136.51 | 234.65 | 45.89 | 20 | 36 | 35 | 39 | 20 |
| 4 | 19.88 | 99.07 | 138.57 | 115.01 | 10.62 | 10 | 27 | 37 | 25 | 16 |
| 5 | 79.56 | 53.47 | 182.65 | 116.76 | 28.74 | 16 | 15 | 18 | 13 | 15 |
| 6 | 104.04 | 19.57 | 264.77 | 16.12 | 209.99 | 19 | 12 | 16 | 4 | 12 |
| 7 | 27.54 | 30.82 | 22.52 | 100.29 | 93.63 | 20 | 6 | 6 | 4 | 18 |
| 8 | 61.98 | 9.12 | 22.8 | 22.58 | 91.58 | 21 | 7 | 5 | 4 | 17 |
| 9 | 28.72 | 3.74 | 42.07 | 0 | 51.2 | 16 | 1 | 5 | 0 | 12 |
| 10 | 23.07 | 0 | 25.79 | 0.001 | 656.98 | 12 | 0 | 3 | 1 | 16 |
| 11 | 12.3 | 0 | 5.32 | 2.44 | 58 | 11 | 0 | 1 | 2 | 13 |
| 12 | 285.74 | 3.29 | 0 | 0 | 311.33 | 203 | 3 | 0 | 0 | 35 |
| Sum | 2717.26 | 2717.26 | 2717.26 | 2717.26 | 2717.26 | 574 | 574 | 574 | 574 | 574 |


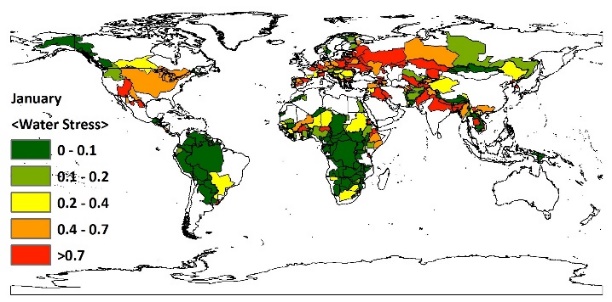

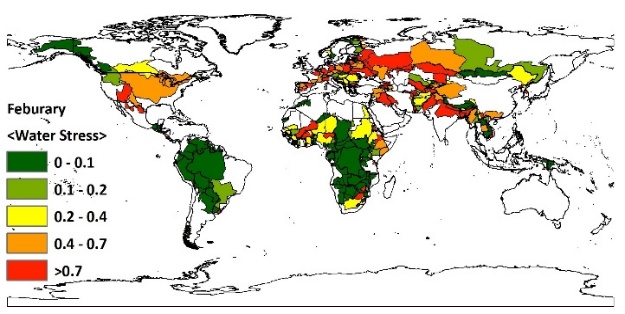


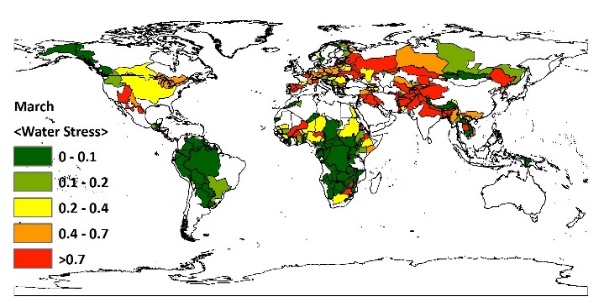

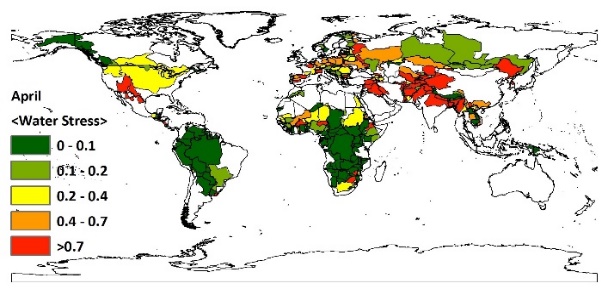


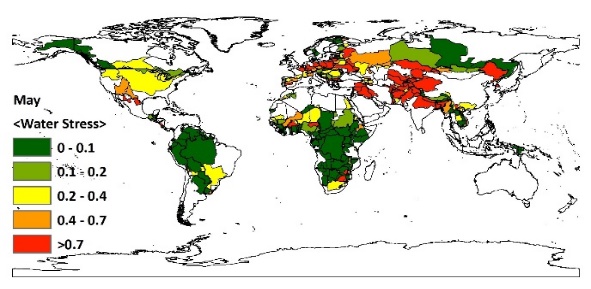

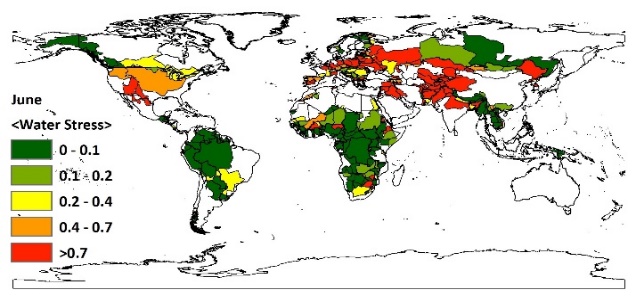


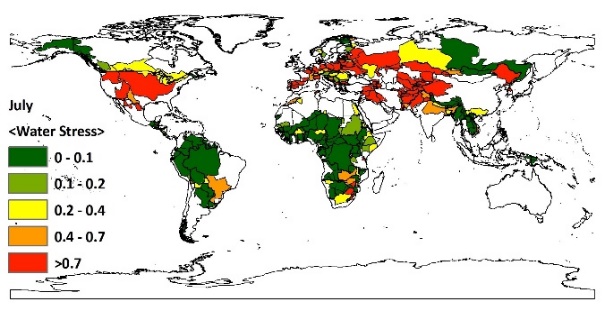

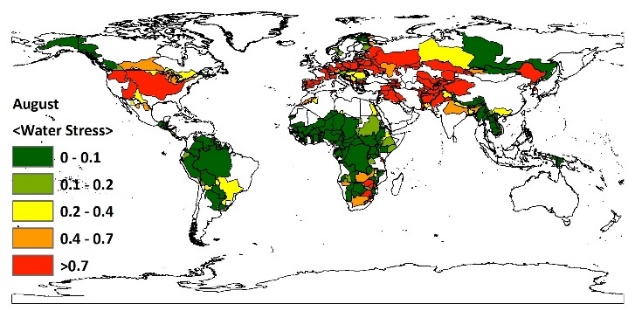


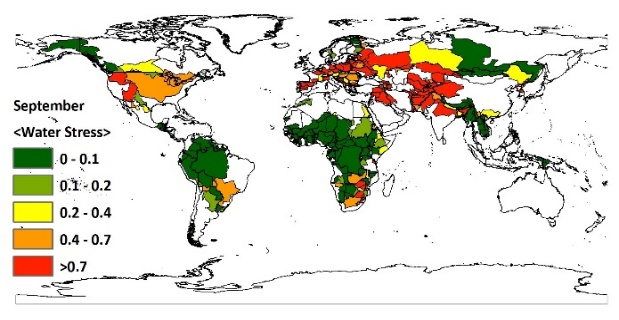

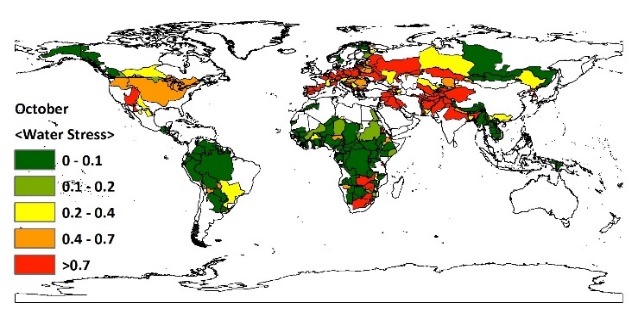


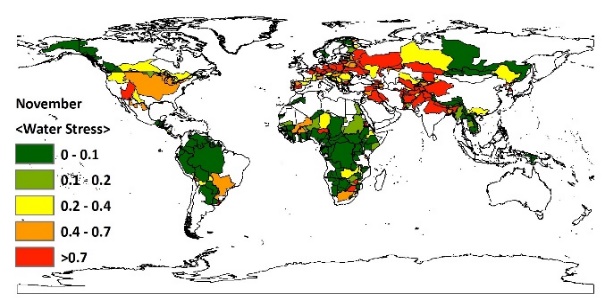

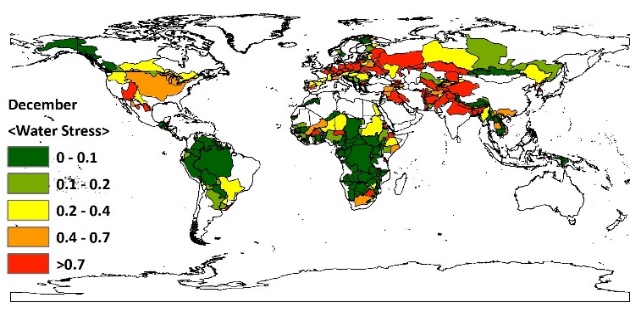


**Figure S1.** Monthly water stress without upstream withdrawal at country-basin unit spatial resolution. This map was generated with ArcGIS 10.2 for desktop from Environmental Systems Research Institute(ESRI) [25].


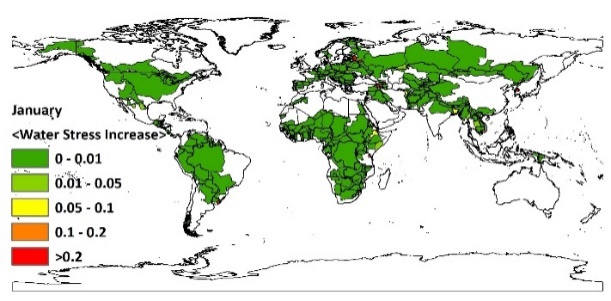

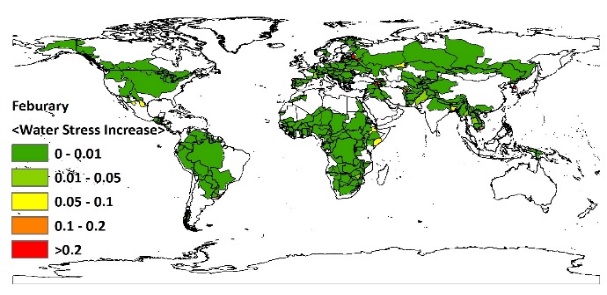


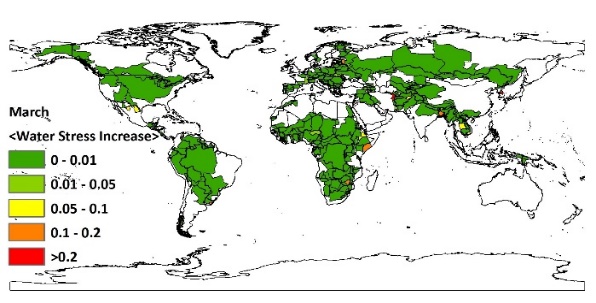

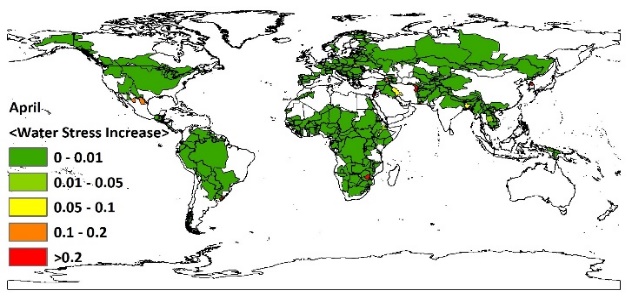


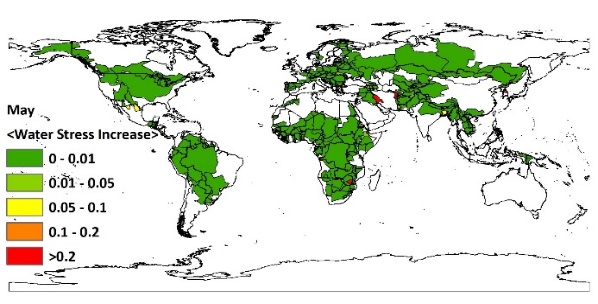

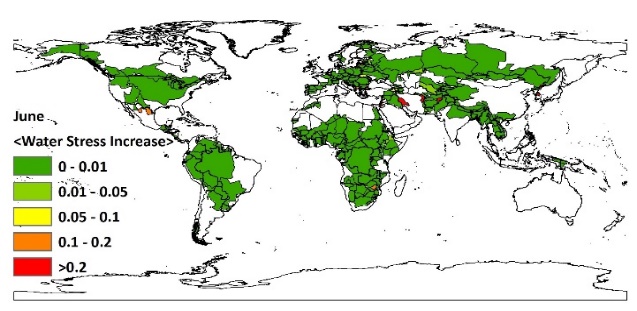


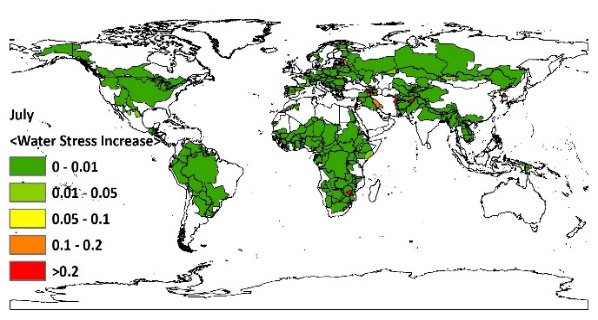

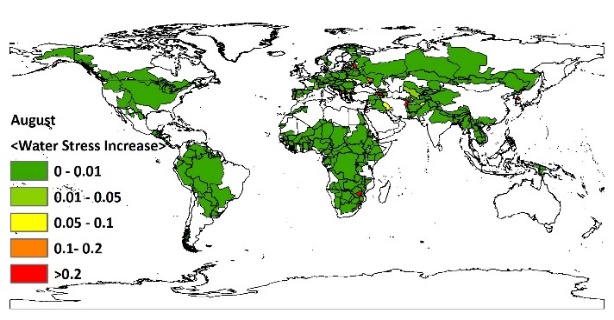


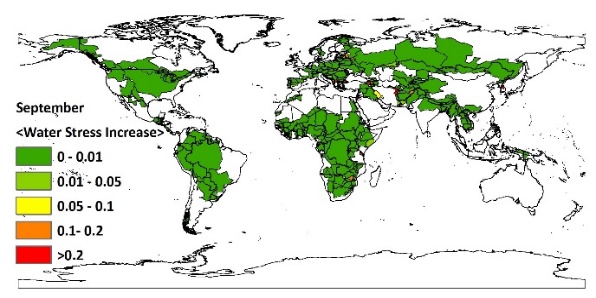

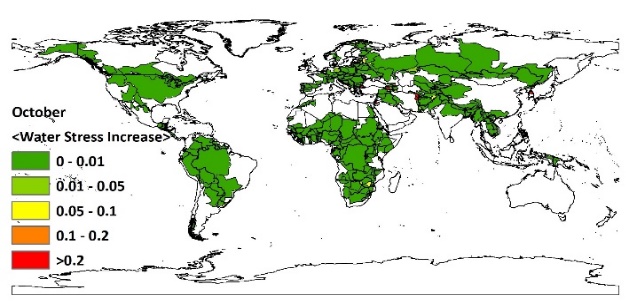


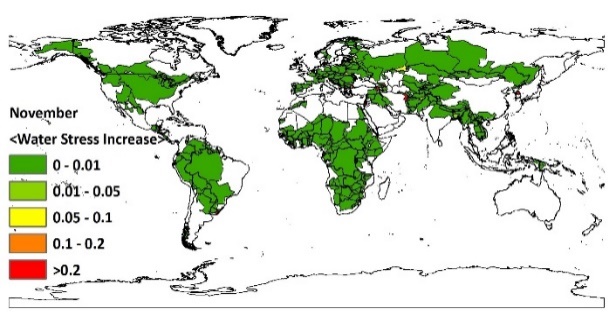

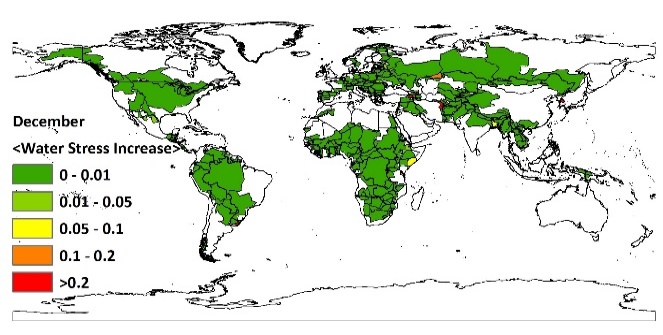


**Figure S2.** Monthly water stress intensification due to upstream withdrawal at country-basin unit spatial resolution. ArcGIS 10.2 for desktop from Environmental Systems Research Institute(ESRI)[25].
