# Supplementary material for: The Impact of Upstream Sub-Basins’ Water Use on Middle Stream and Downstream Sub-Basins’ Water Security at Country-Basin Unit Spatial Scale and Monthly Temporal Resolution
Source: Int J Environ Res Public Health. 2019 Feb 3;16(3):450. doi: 10.3390/ijerph16030450 (PMC6388158; doi:10.3390/ijerph16030450)
Supplement: Supplementary file 1 [file ijerph-16-00450-s001.zip › ijerph-430587 - supplementary -proofed/Supplementary Material.pdf]

Supplementary Material

Table S1. Number of people (in millions) and a number of country-basin units facing different levels of water stress. UN-adjusted population count for the target year (2010) obtained from the Center for International Earth Science Information Network (CIESIN)17 was used.

Number of Months Per Year (n)	Number of People (in Millions) Facing Low, Moderate, Significant and Severe Water Scarcity during n Months Per Year					Number of Country-Basin Units Facing Low, Moderate, Significant and Severe Water Scarcity during n Months Per Year				
	**0–0.1**	**0.1–0.2**	**0.2–0.4**	**0.4–0.7**	**>0.7**	**0–0.1**	**0.1–0.2**	**0.2–0.4**	**0.4–0.7**	**>0.7**
0	1953.41	1934.7	1484.02	1051.17	953.45	192	339	335	378	368
1	27.15	249.34	245.51	323.65	77.29	22	67	71	57	15
2	59.11	122.18	148.73	734.59	128.55	12	61	42	47	17
3	34.76	1191.97	136.51	234.65	45.89	20	36	35	39	20
4	19.88	99.07	138.57	115.01	10.62	10	27	37	25	16
5	79.56	53.47	182.65	116.76	28.74	16	15	18	13	15
6	104.04	19.57	264.77	16.12	209.99	19	12	16	4	12
7	27.54	30.82	22.52	100.29	93.63	20	6	6	4	18
8	61.98	9.12	22.8	22.58	91.58	21	7	5	4	17
9	28.72	3.74	42.07	0	51.2	16	1	5	0	12
10	23.07	0	25.79	0.001	656.98	12	0	3	1	16
11	12.3	0	5.32	2.44	58	11	0	1	2	13
12	285.74	3.29	0	0	311.33	203	3	0	0	35
Sum	2717.26	2717.26	2717.26	2717.26	2717.26	574	574	574	574	574

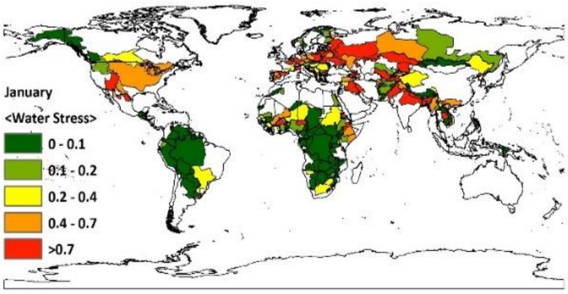

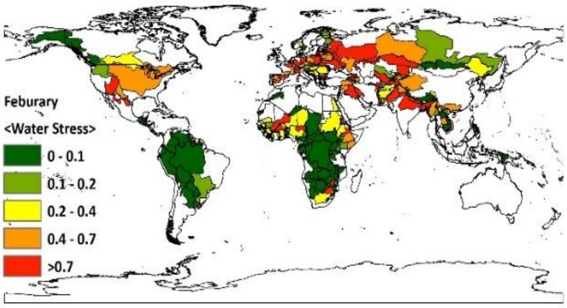

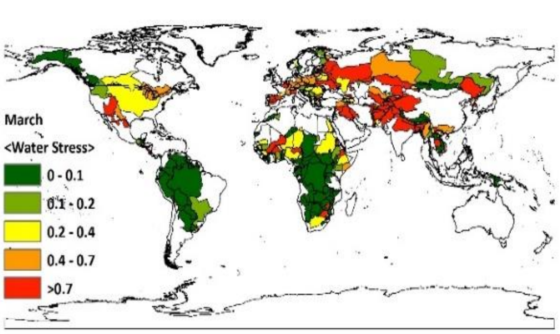

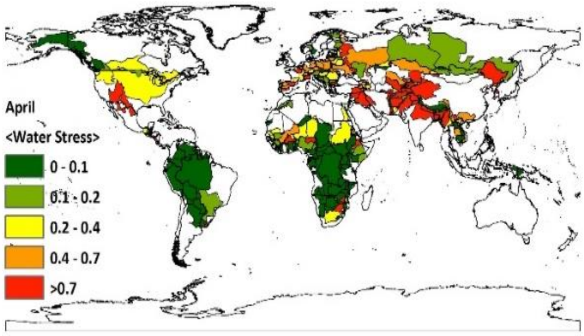

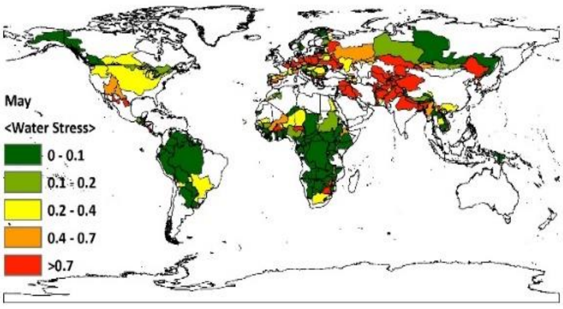

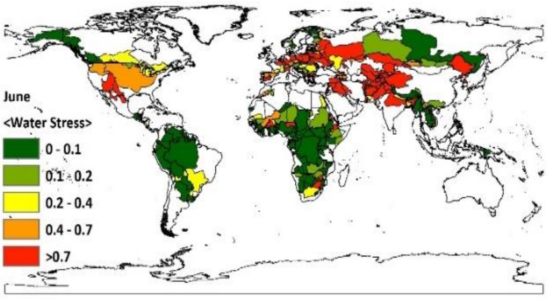

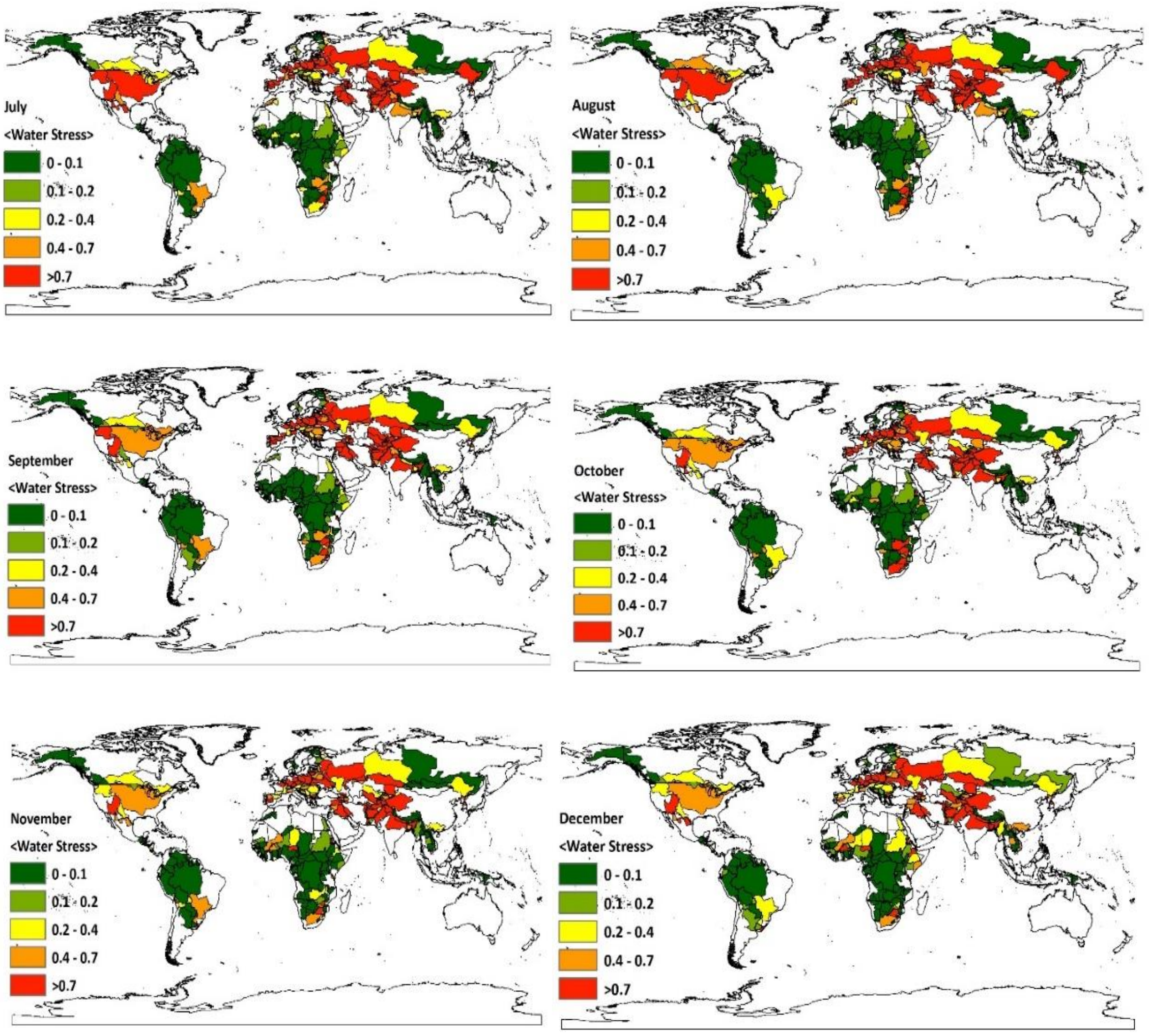

Figure S1. Monthly water stress without upstream withdrawal at country-basin unit spatial resolution. This map was generated with ArcGIS 10.2 for desktop from Environmental Systems Research Institute(ESRI) [25].

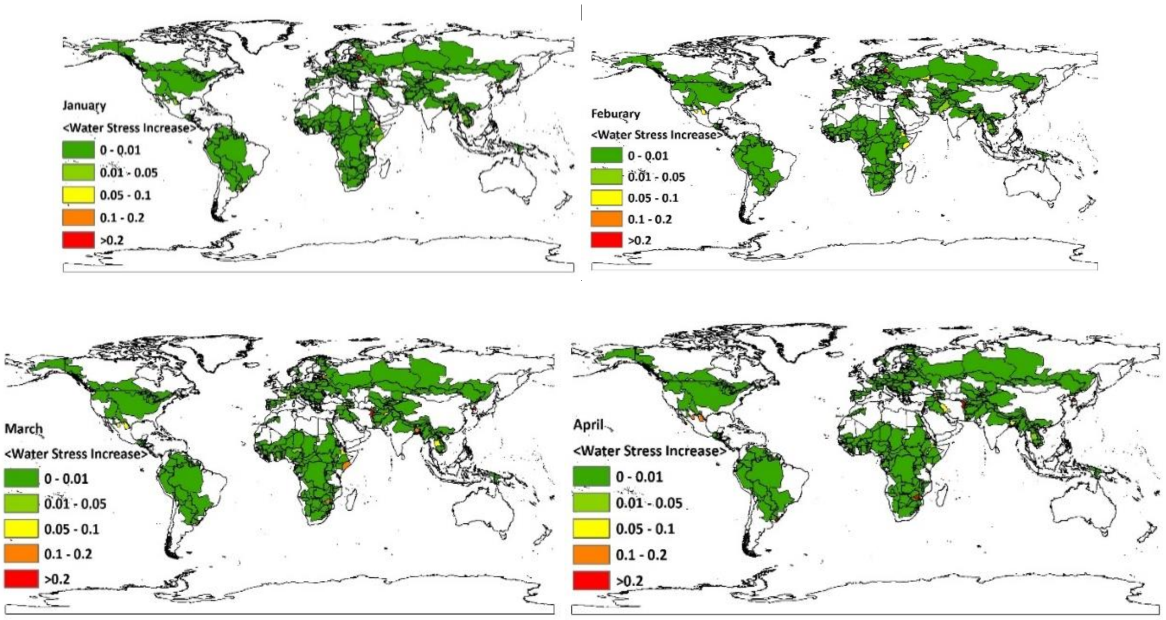

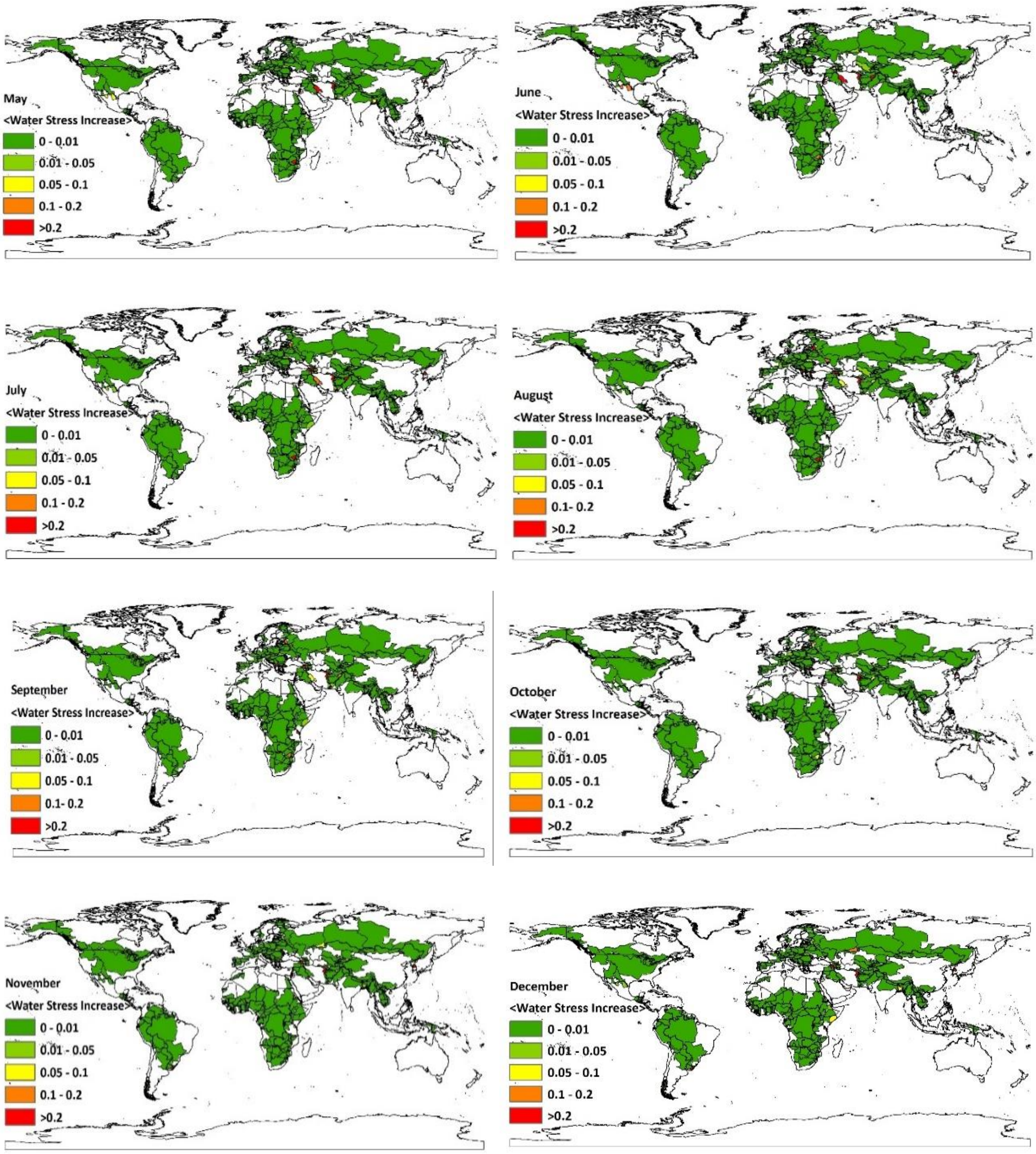

Figure S2. Monthly water stress intensification due to upstream withdrawal at country-basin unit spatial resolution. ArcGIS 10.2 for desktop from Environmental Systems Research Institute(ESRI)[25].
